# Acquired reactive perforating collagenosis secondary to Cabozantinib: A case report and literature review

**DOI:** 10.1177/2050313X241304961

**Published:** 2024-12-16

**Authors:** Zahra Rehan, Andrea Haner, Amina Taleb, Nzechukwu Ikeri, Jori Hardin

**Affiliations:** 1 Cumming School of Medicine, University of Calgary, Calgary, AB, Canada; 2 The Jack Ady Cancer Centre, Lethbridge, AB, Canada; 3 Chinook Regional Hospital, Lethbridge, AB, Canada; 4 Division of Dermatology, University of Calgary, Calgary, AB, Canada

**Keywords:** general dermatology, medical dermatology, drug response, drug reactions, reactive perforating collagenosis, acquired perforating dermatosis

## Abstract

Acquired perforating dermatoses (APD) encompass a group of skin conditions distinguished by transepidermal elimination of dermal components. Acquired reactive perforating collagenosis (ARPC), a subtype of APD, has been reported most commonly in association with diabetes mellitus, chronic renal failure, and medications. In this report, we identify a novel case of ARPC secondary to Cabozantinib treatment.

## Introduction

Acquired perforating dermatoses (APD) encompass a group of skin conditions distinguished by transepidermal elimination of dermal components such as collagen, elastic tissue, or necrotic connective tissue. APD can be categorized into primary and secondary forms. The four classic forms of primary perforating dermatoses are familial reactive perforating collagenosis (RPC), elastosis perforans serpiginosa (EPS), perforating folliculitis (PF), and APD, also known as Kyrle disease.^1^ RPC can be inherited in childhood or acquired in adulthood, and is characterized by transepidermal elimination of altered collagen through the epidermis.^2^ Acquired reactive perforating collagenosis (ARPC) is an uncommon cutaneous presentation seen in association with diabetes mellitus (DM), chronic renal failure (CRF), malignancy, and medications.^3^ Several case reports document ARPC associated with tyrosine kinase inhibitors (TKIs).^10,12–14,16^ Herein, we report a patient who developed ARPC after starting Cabozantinib therapy. To our knowledge, this is the first report of ARPC secondary to Cabozantinib.

## Case Report

**Figure 1.**
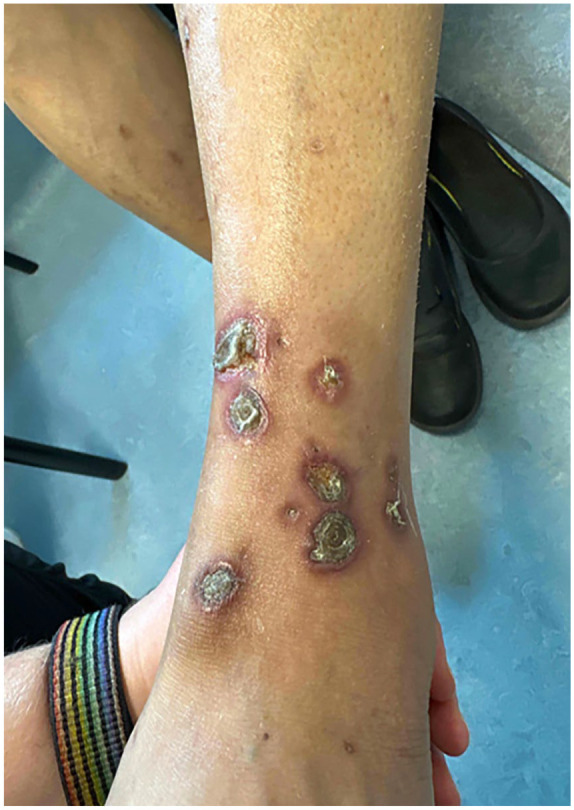
Crusted punched out ulcers with central eschar and peripheral rim of erythema on the lower leg.

**Figure 2.**
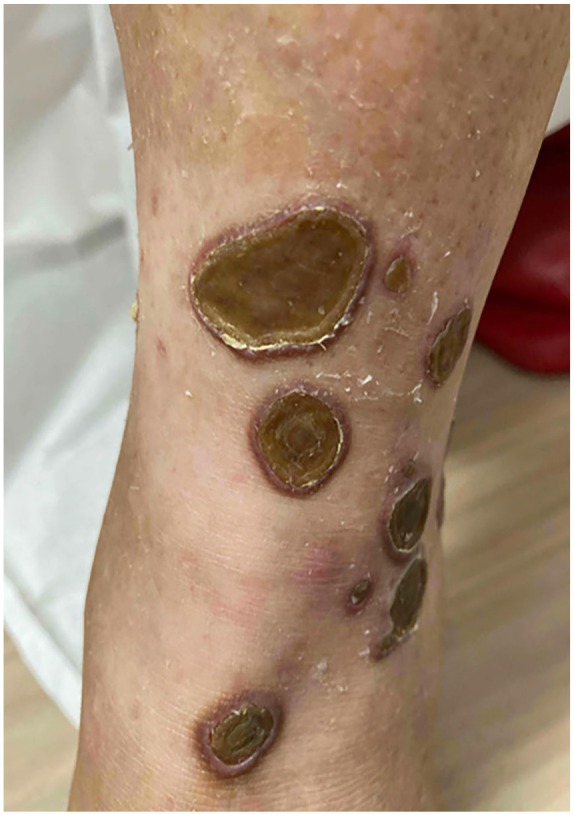
Progression to larger ulcers with thick yellow-brown eschar and diminished peripheral erythema.

**Figure 3.**
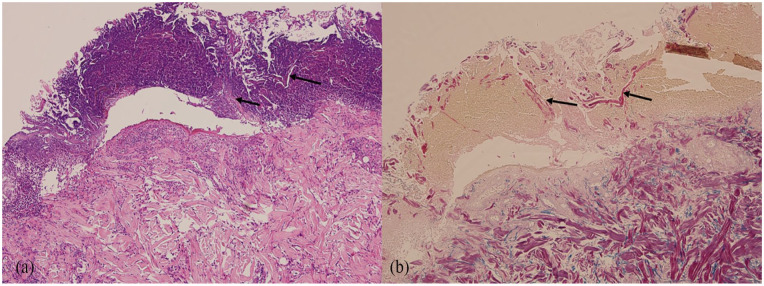
(a) Hematoxylin & Eosin ×100 showing transepidermal elimination of collagen fibers (arrows) into the overlying inflammatory crust. (b) Massons Trichrome ×100 shows red collagen fibers (arrows) within the crust.

A 60-year-old female with a diagnosis of metastatic clear cell carcinoma of the kidney presented with a 5-month history of superficial ulcers on the lower legs with overlying thickened eschar. The patient had a history of longstanding diabetes with a recent HbA1c of 6.8. Eight months prior, she started Cabozantinib therapy for her diagnosis of metastatic cancer. There were no new medications in her profile and diabetes was longstanding and controlled to target with Insulin and Janumet. Initially, she was prescribed topical Fucidin 2% ointment, topical Betaderm 0.1% cream, and a 5-day course of prednisone, all of which provided minimal relief. Bacterial swabs for culture and sensitivity showed 4+ gram-positive cocci but the culture grew *Klebsiella pneumonia* complex. Subsequently, she was treated with multiple courses of antibiotics including Cephalexin, Amoxicillin–Clavulanate, and Ciprofloxacin. These agents decreased pain and peripheral erythema of the lesions but did not result in improvement of the ulcers. On presentation to the dermatology clinic, multiple, well-demarcated circular to ovoid ulcers with raised, erythematous to violaceous borders, and adherent yellow-brown overlying eschars were noted on the bilateral lower legs (Figures 1 and 2). Histology revealed transepidermal elimination of collagen on Masson’s trichrome stain, and inflammatory cellular debris, in keeping with a diagnosis of RPC (Figure 3). Cabozantinib was held. A trial of Tazarotene 0.045% was initiated with the patient noting improvement. A retrial of Cabozantinib resulted in worsening of skin and the development of new ulcers, and as a result, this treatment was stopped indefinitely. At 3-month follow-up, the lesions were healing slowly with continued Tazarotene 0.045% and drug withdrawal.

## Discussion

RPC is a rare perforating dermatosis in which altered collagen bundles extrude through the epidermis. This reaction is usually associated with inflammatory, malignant, or systemic diseases, most commonly DM and CRF. Less often, ARPC has been linked with hepatic dysfunction, thyroid dysfunction, viral infections, and cancers.^1^ The exact pathogenesis of ARPC is unknown but there are several hypotheses. One theory is that chronic scratching leads to damage of the epidermis or dermal collagen, eventually causing the onset of ARPC.^1,4^ Patients with APD often exhibit the Koebner phenomenon, where onset of new skin lesions develops at sites of trauma.^1^ An alternative theory suggests a genetic anomaly in collagen is responsible for focal damage, resulting in collagen perforation following necrolysis of the overlying epidermis.^4^

We reviewed cases of ARPC reported in the literature associated with medications (Table 1). A common thread across all cases involved the presentation of pruritic lesions (17/20) and the onset of manifestations following initiation of treatment for cancer (11/20). The clinical appearance was described as round to oval umbilicated papules or nodules and crusted ulcers with central keratotic plug.^1^ The papules can start very small and gradually progress to 5–6 mm in diameter, featuring a concave center.^4^ The lesion distribution was predominantly noted on the extremities (16/20), followed by the trunk (5/20) and face (5/20). The most common histopathology showed the presence of cup-shaped invagination in the epidermis (13/20), inflammatory infiltrate (14/20), and transepidermal elimination of collagen bundles (17/20). The histopathologic features of ARPC are not uniform and may resemble any of the four classic primary perforating dermatoses (RPC, EPS, PF, and APD).^1^ Multiple biopsies are sometimes required before establishing a definitive diagnosis in which elimination of collagen fibers into the epidermis is observed.^4^ Faver et al. introduced the following diagnostic criteria for ARPC: onset of condition after the age of 18, presence of umbilicated nodules or papules with a central hyperkeratotic plug, and transepidermal elimination of necrotic collagen tissue.^5^ In our review, monoclonal antibodies (9/21) emerged as the predominant drug category associated with ARPC reactions, followed by TKIs (5/21) and protease inhibitors (3/21). We did not find any case reports of ARPC reactions associated with Cabozantinib therapy.

**Table 1.** A literature review of case reports documenting acquired reactive perforating collagenosis associated with medication use.

Case	Age/Sex	Associated drug	Presentation	Clinical appearance	Distribution	Histology	Treatment
1	59/M	Nivolumab + Ipilimumab	Eruption of pruritic nodules that appeared 4 weeks after starting treatment with nivolumab and ipilimumab for renal cancer^7^	Diffuse coalesced erythema and few red papulonodules followed by an increase in reddish papulonodules presenting with central ulcerative umbilication	Trunk and extremities	Necrosis of the epidermis with transepidermal elimination of degenerated collagen fibers	Narrow-band UVB narrow-band ultraviolet light B (NB-UVB) phototherapy
2	62/F	Panitumumab	Pruritic papular lesions that appeared after the sixth cycle of panitumumab for advanced colon cancer^6^	Round to oval erythematous papules with collapsed centers and keratotic plugs	Back and extremities	Cup-shaped invagination plugged by basophilic collagen, necrotic inflammatory cells, and degenerative collagen fibers passing through the epidermis vertically	Cessation of panitumumab
3	73/M	Ranibizumab	Generalized, excessively pruritic rash that appeared 1 month after starting treatment with intravitreal ranibizumab injections to the right eye for occult choroidal neovascular membrane^11^	Generalized, erythematous rash with umbilicated papules with ridge-like borders and keratin plugs	Trunk, lumbar region, and extensor aspects of the upper and lower extremities	Cup-shaped depression of the epidermis associated with a keratin plug containing compact orthokeratosis and parakeratosis with granular nuclear debris, and altered collagen fibers in the underlying dermis, with focal extrusion through the epidermis	Switch therapy to aflibercept and started NB-UVB phototherapy
4	68/M	Erlotinib	Two-month history of pruritic, red lesions that appeared 2 weeks after starting erlotinib therapy for metastasized small cell lung carcinoma^13^	Nodules with keratotic plugs on upper back and shoulders, numerous acneiform red papules and pustules on the trunk, and periungual granuloma on second and fourth digits of right hand, and third digit on left hand	Scalp, face, back and trunk	Cup-shaped depression in the epidermis, transepidermal elimination of basophilic collagen bundles, surrounded by a lymphocytic and neutrophilic infiltration, and degenerated collagen fibers transversely eliminated through the epidermis	Dose decrease of erlotinib and topical betamethasone butyrate propionate
5	53/F	Erlotinib	Two-month history of skin eruptions and pruritic, red lesions that appeared 1 week after starting erlotinib therapy for lung adenocarcinoma^14^	Scattered or densely distributed erythematous follicular papules and papulopustules involving the face, neck, and upper trunk, and multiple erythematous, umbilicated, dome-shaped papules noted on the trunk and extremities, a few of them partially coalescing to form plaques	Face, neck, trunk, buttocks, perineum, and extremities	Cup-shaped depression of the epidermis with a keratin plug showing parakeratosis, inflammatory debris, degenerated collagen fibers, and perivascular infiltrate of inflammatory cells below the depression, along with elimination of degenerated collagen fibers through the epidermis	Oral isotretinoin 10 mg, topical mometasone furoate cream 0.1% and tretinoin cream 0.1%, cessation of erlotinib
6	83/M	Sorafenib	Ten-month history of intensely pruritic skin lesions that appeared 11 months after starting treatment with sorafenib for hepatocellular carcinoma^10^	Crater-shaped, erythematous papulonodules with hyperkeratotic centers, some with secondary excoriations from scratching	Abdomen	Extensive epidermal ulceration with underlying verticalized collagen fibers	Oral antihistamine and topical corticosteroid therapy
7	65/M	Sorafenib	One-year history of painful, pruritic lesions that presented 2 weeks after starting treatment with sorafenib for hepatocellular carcinoma^12^	Solitary and grouped, brown to slightly erythematous papules with a central hyperkeratotic, depressed core scattered on the thighs, knees, and left elbow, along with hyperpigmented papulonodules with a central keratotic core on the buttocks and several flesh-colored papules on the scrotum	Lower extremities, upper extremities, buttocks and scrotum	Epidermal invagination surrounding and forming a channel for aggregates comprised of eosinophilic fibers, neutrophils, and basophilic debris, and an overlying parakeratotic core with adjacent epidermal hyperplasia	*Not listed*
8	50/M	Telaprevir	Pruritic ulcerated lesions that presented 3 weeks after starting treatment with telaprevir for hepatitis C viral infection^15^	Extensive xerosis and ulcerated papular and nodular lesions with an inflammatory border and a central keratotic plug	Lower legs	Cup-shaped depression in the epidermis covered by a hyperkeratotic crust containing necrotic debris, inflammatory cells, and collagen bundles oriented perpendicularly to the surface and extruded through the epidermis	Cessation of telaprevir, initiating betamethasone 0.05%? dipropionate and petroleum jelly daily
9	46/M	Gefitinib	Facial acneiform eruption that appeared 2 weeks after starting treatment with gefitinib for squamous cell carcinoma of the lung, followed by 2 month history of red and pruritic lesions that appeared on the legs^16^	Erythematous nodules, discreetly infiltrated on palpation and centered by a hyperkeratotic scab with underlying ulceration	Lateral aspect of both thighs	Epithelium with acanthosis and hyperkeratosis, with a central invagination including orthokeratotic and parakeratotic keratin, inflammatory cells and necrotic debris, along with collagen bundles arranged vertically crossing the epidermis	Cessation of gefitinib and topical emollients
10	51/M	Bevacizumab (5 mg/kg every 2 weeks)	Two-month history of pruritic lesions that appeared 2 weeks after starting treatment with bevacizumab for metastasized colorectal cancer^17^	Erythematous, umbilicated papules with adherent, central keratotic plugs	Posterior and lateral aspects of the neck	Irregular acanthosis of the epidermis with accumulation of basophilic collagen in the dermal papillae and a keratotic corneal plug, and collagen bundles in vertical orientation within the epidermis	Topical corticosteroids
11	55/M	Natalizumab	Eleven-month history of widespread pruritic eczematous eruption that appeared five years after starting treatment with natalizumab for relapse of multiple sclerosis^18^	Bilateral, symmetrical, well-defined plaques of perforating papules and nodules on a background of erythematous skin	Posterior arms and legs	Shallow, cup-shaped disruptions of acanthotic epidermis with thick plugs of basophilic debris and collagen elimination to the surface	Switch to Ocrelizumab and initiated NB-UVB phototherapy
12	43/F	Natalizumab (300 mg/month IV)	Pruritic eruption and umbilicated papules that appeared 6 months after starting treatment with natalizumab for relapsing-remitting multiple sclerosis^19^	Erythematous umbilicated papules, some of which had a central depression and others contained a keratotic plug	Lower legs and ears	Intense eosinophilic collagen mixed with neutrophil debris intermingled with keratinocytes	Emollients, topical corticosteroids and oral corticosteroids
13	56/F	Cetuximab	Cutaneous eruption with intense pruritus that appeared 1 month after starting chemotherapy (cetuximab) for metastasized spindle cell carcinoma of the tongue^20^	2–3-mm round to oval erythematous keratotic papules and nodules with centered yellow to black crusts	Face and distal four extremities	Cup-shaped invagination with basophilic debris and underlying upper dermal predominant inflammatory cell infiltration, and transepidermal elimination of collagen fibers into the basophilic debris	Cessation of paclitaxel and cetuximab
14	53/M	Necitumumab	Three-month history of acneiform eruptions that appeared 2 weeks after starting treatment with necitumumab for stage 3 non-small-cell lung cancer^21^	Disseminated papules and nodules with central yellow–green crust-like structures and small acneiform pustules, with no keratotic follicular-based papules, pruritus, or excoriations observed	Lower leg with gradual spread to whole body	Cup-shaped ulceration with abundant neutrophil infiltration and transepidermal elimination of degenerated collagen fibers	Cessation of necitumumab
15	40/F	Leflunomide (10 mg/d)	Acute, red, and pruritic eruption that appeared 6 months after starting treatment with leflunomide for cutaneous small cell vasculitis^23^	Erythematous papules with central crusting	Upper and lower extremities	Cup-shaped invagination of the epidermis with areas of collagen intercalating and between the keratinocytes, with areas of necrotic, inflammatory debris	Cessation of leflunomide
16	26/F	Azathioprine	Slightly pruritic lesions on the hands that appeared 3 weeks after starting treatment with azathioprine for management of ulcerative pancolitis^24^	5–10 mm erythematous and umbilicated papules, with adherent central keratotic plug	Dorsum of the hands and lateral aspects of the neck	Superficial dermal necrosis, with transepidermal elimination of verticalized collagenous fibers and necrotic debris	Switched azathioprine to mesalazine and initiated topical tacrolimus ointment 0.1%
17	51/F	Sirolimus (2.5 mg/day)	Four-month history of acneiform eruption of the face that appeared 3 weeks after starting treatment with sirolimus for impaired renal function^8^	Multiple slightly tender erythematous papulonodular lesions on the forehead, cheeks, upper back, and thorax, along with less erythematous, nontender, flattened lesions with a centered crust or plug	Seborrheic parts of face and upper trunk	Predominantly lymphocytic infiltrate with well-demarcated perivascular and perifollicular distribution	Hydroxychloroquine (200 mg/day) without modification of other medications
18	40/M	Indinavir	Pruritic lesions with diffuse dryness that appeared 3 weeks after starting indinavir treatment for HIV+ status^9^	Widespread ulcerated papules with diffuse underlying xerosis	Extensor aspect of the left leg	Cup-shaped depression in the epidermis, containing necrotic debris and a mixed chronic inflammatory infiltrate; in the dermis, basophilic collagen bundles were oriented perpendicularly to the surface and perforated the epidermis	Topical corticosteroids, UVB-NB, cessation of indinavir and replaced with nelfinavir
19	29/M	Indinavir	Painful lesions and diffuse dryness that appeared 5 weeks after starting indinavir treatment for AIDS^9^	Multiple excoriated, ulcerated, and pruritic papules	Four limbs	Mixed chronic inflammatory infiltrate with basophilic collagen bundles oriented perpendicularly to the surface, transgressing the epidermis	Cessation of indinavir
20	36/M	Terepril	Localized, itchy eruptions that appeared 15 weeks after starting treatment with terepril for metastasized sigmoid colon adenocarcinoma^22^	Multiple discrete erythematous papules and nodules with central keratotic plugs on the right lower leg and coalesced plaques extending in a linear distribution on the right heel	Right lower extremity and heel	Epidermal invaginations filled with keratin, epithelium at the base of the invagination revealed parakeratosis, interface change, and focal rupture of the epithelium, with keratotic debris perforating into upper dermis and associated lymphohistiocytic infiltrate	Initiating halometasone cream 0.05% and cessation of terepril

Several case reports have documented ARPC secondary to initiating therapy with TKIs.^10,12–14,16^ Some authors suspect a preceding history of DM as an exacerbation or triggering factor for the onset of ARPC, as was seen in our case. However, in these cases, DM was well-controlled with laboratory investigations that revealed glucose and HbA1c values within normal limits.^12,13^ Other authors have reported a preceding diagnosis of carcinoma, including hepatocellular carcinoma, small cell lung carcinoma, squamous cell carcinoma of the lung, and lung adenocarcinoma.^12–14,16^ However, Fernández-Guarino and Suzuki link the onset of the cutaneous manifestations after initiating Gefitinib and Erlotinib therapy for cancer.^13,16^ ARPC has also been associated with monoclonal antibodies and protease inhibitors (Table 1). Differentiating whether the malignancy or treatment of malignancy is the primary association is challenging as a wide range of onset of the reaction to drug initiation vs. cancer diagnosis has been documented. In our case report, the onset of reaction to drug initiation was 12 weeks, slightly higher than the average of 7 weeks in our literature review (Table 1).

Currently, there are no established management guidelines for ARPC and proposed treatments are based on case reports.^1^ In patients with APD, the goal of therapy is to treat the underlying disease and address the symptoms associated with the reactions. The first-line treatment strategy includes topical corticosteroids, topical retinoids, and topical keratolytic agents such as urea or salicylic acid.^1^ The addition of topical emollients and oral antihistamines aims to relieve pruritus. Combination therapy of topical corticosteroids with topical retinoids, antihistamines, or topical antibiotics may show more favorable outcomes rather than monotherapy alone.^25^ In addition to topical therapy, systemic treatment with oral antibiotics and narrow-band UVB phototherapy should be considered, depending on the underlying primary disease.^25^

In our patient, topical Tazarotene 0.45% and cessation of Cabozantinib led to gradual improvements after failing topical corticosteroids and antibiotics, prednisone, and oral antibiotics. In conclusion, ARPD is an uncommon condition associated with systemic diseases and certain classes of medications. We report a novel and uncommon case of ARPC linked to Cabozantinib therapy, demonstrating partial improvement with topical Tazarotene 0.045% and discontinuation of the implicated medication.
